# Editorial: Highlights in sports management, marketing and business

**DOI:** 10.3389/fspor.2023.1161143

**Published:** 2023-02-27

**Authors:** Hans Westerbeek, Rochelle Eime

**Affiliations:** ^1^ Institute for Sport and Health, Victoria University, Melbourne, VIC, Australia; ^2^ School of Health and Life Sciences, Federation University Australia, Ballarat, VIC, Australia

**Keywords:** sport marketing, sport management, sport business, governance, major events

**Editorial on the Research Topic** Highlights in sports management, marketing and business

In this research topic we aim to provide an insight into the wide range of topics that are of interest in the context of the business of sport. Whilst specifying sport management and sport marketing in the title of the research topic, the unifying context really is the “business of sport”. This ranges from what traditionally has been perceived as sport business – professional sport and all that comes with fan engagement, including spectating and social media for elite sport, through to the grassroots of community sport participation, which is a different form of “sport business”.

The collection of articles in this topic showcase this varied, international and diverse range of sport business insights that can be delivered through innovative and cutting-edge research. Several of the studies focus on the Olympic Games as well as other international events and sporting organisations, which may be an indication that elite sports (events) remain an attractive target of sport business research. We will briefly introduce each of these projects in the next section.

Philippou and Hines focus on what are the best practice policies in Anti-Bribery and Corruption (ABC) policies. Undertaking a critical review of the diverse ABC governance policies in 22 of the largest International Sports Governing Bodies (ISGBs) through content analysis on governance documents publicly available. They report that, although ISGBs are improving, there remains an absence of (industry wide) adequate policies in regard to managing the risk of bribery. They recommend sharing best practices and for an external enforcement organisation to provide guidance on ABC policies for ISGBs.

Gallant and Belanger, in a scoping review, explored what empirical evidence is there that supports sport participation and physical activity-based models along the journey of an individual's participation in sport. They reviewed 17 different sport participation models. They concluded that most supporting evidence came from models that focused on elite level athletes but in order to provide evidence-based input into sport programs, policies and practices, more population-based research was required.

Wang, Gao and Wang provide a perspective on a commercial dilemma of fitness clubs – how the construct of “commercial friendship”, mediates the intent to repurchase. Commercial friendship refers to a level of trust and friendship between the service provider and customer. They report that leisure involvement had a positive impact on repurchase intention and satisfaction and that there was a mediating effect of commercial friendship in the relationship between leisure involvement, repurchase intention, and satisfaction.

Rocha and Xiao conducted a systematic review on the displacement of host community residents when sport mega events were hosted. It was reported that displacement of residents has been studied exclusively in the context of the Olympic Games, since Seoul 1988. The size and scope of the event explains why the Olympics has been frequently associated with resident displacement. The authors reported that residents suffered either direct, forced evictions or indirect displacements. An important finding is that the narratives produced by sport mega-events guardians that argue an alignment with the United Nations Sustainable Goals (SDG) contradict the actual practice in relation to ensuring human rights within host cities of such events.

Looking at similar issues in regard to human rights and mega events, O'Rourke and Theodoraki focus on the Qatar World Cup and its sustainability strategy from the perspective of three key network stakeholders – FIFA, the Federation of International Football Associations, Qatar's Supreme Council (SC), and the Local Organising Committee (Q22). A Qualitative Content Analysis of documents delivered a range of insights that led the authors to conclude that the tripartite policy network of actors (FIFA, SC and Q22) in their governance approach delivered some cohesive policy formulation, had varied resources at their disposal, that there were inconsistencies in accountability measures and that the lead network role was dependent on specific actor initiatives and commitments.

In another scoping review, Bodin, Teare and Taks acted on the expressed need to broaden the scope of research in sport management, in this case, to include critical social science.

They found that since 2005, there is a noticeable increase in critical research published in sport management journals but that there remains a relatively low number of critical social science insights published in sport management journals. They also argue that researchers could better articulate their research approaches in scholarly outputs.

Grabmüllerová explored whether social media content produced by National Olympic Committees (NOCs) during the 2020 Tokyo Olympic Games had any influence on the International Olympic Committee's (IOC) gender equality ambitions. She reported that media personnel in particular, have great influence on the framing of gender identities and stereotypes in their communication channels. Grabmuüllerová argues that in contrast to news media, the NOCs knew the framing they were applying, and they did it in alignment with the Olympic values which led to more equal representation of male and female athletes.

Also, in context of the 2020 Tokyo Olympic Games, Dittmore and Kim found that nations and media outlets use various approaches to creating and interpreting the medal tables of the Olympics. The often created a narrative that presented the best Nationalistic picture. American media in particular often presented an overview that would place the USA on top of the table, even when China had won more gold medals. In non-American media, this approach was often criticized.

In a scoping review conducted by Durden-Myers and Swaithes, they investigated whether or not free access to opportunities to participate in sport and physical activity devalued participation. Their focus was on children and young adults. They found that subsidies to participate or free offers could improve participation generally and also be effective in attracting those from lower socio-economic backgrounds. A major challenge is presented by the fact that this effect disappears with increasing deprivation of the target population. Hence groups with the highest levels of deprivation will have wider complexities to deal with that are affecting their participation. Competing priorities and potentially unrealistic expectations of stakeholders were also identified as issues.

In regard to the dominance of sport governance models and practices by the “Global North” countries, García and Meier explore the extent that Global South countries have a sense of autonomy when it comes to developing their sport governance structures. In particular, the research sought to identify the extent to which the International Olympic Committee is successful in implementing the norms and regulations on sports autonomy as a governance transplant, at the national level in countries from the Global South that are part of the Olympic Movement. National structures and legacies have an impact on the way in which the autonomy of sport is translated in Global South countries but that translational and cultural tensions lead to tensions between stakeholders at the level of government and sport.

Finally, Ruwuya, Juma and Woolf provide a perspective on the challenges that come with implementing anti-doping policy and programs in Africa. They contextualise this perspective by explaining that Africa's views and systems were not specifically considered during the creation of the World Anti-Doping Agency (WADA), which relegated African states to a passive role in global anti-doping monitoring, but subject to the strict compliance requirements for WADA's global policy. Human capacity development and a legacy of colonialism present further contextual challenges to establishing anti-doping support structures and implementing the universal policy.

